# Convolutional Neural Network Incorporating Multiple Attention Mechanisms for MRI Classification of Lumbar Spinal Stenosis

**DOI:** 10.3390/bioengineering11101021

**Published:** 2024-10-13

**Authors:** Juncai Lin, Honglai Zhang, Hongcai Shang

**Affiliations:** 1 School of Medical Information Engineering, Guangzhou University of Chinese Medicine, Guangzhou 510006, China; 2 Dongfang Hospital, Beijing University of Chinese Medicine, Beijing 100078, China; 3 Key Laboratory of Chinese Internal Medicine of Ministry of Education, Beijing University of Chinese Medicine, Beijing 100700, China

**Keywords:** lumbar spinal stenosis, deep learning, attention mechanisms, medical image analysis

## Abstract

Background: Lumbar spinal stenosis (LSS) is a common cause of low back pain, especially in the elderly, and accurate diagnosis is critical for effective treatment. However, manual diagnosis using MRI images is time consuming and subjective, leading to a need for automated methods. Objective: This study aims to develop a convolutional neural network (CNN)-based deep learning model integrated with multiple attention mechanisms to improve the accuracy and robustness of LSS classification via MRI images. Methods: The proposed model is trained on a standardized MRI dataset sourced from multiple institutions, encompassing various lumbar degenerative conditions. During preprocessing, techniques such as image normalization and data augmentation are employed to enhance the model’s performance. The network incorporates a Multi-Headed Self-Attention Module, a Slot Attention Module, and a Channel and Spatial Attention Module, each contributing to better feature extraction and classification. Results: The model achieved 95.2% classification accuracy, 94.7% precision, 94.3% recall, and 94.5% F1 score on the validation set. Ablation experiments confirmed the significant impact of the attention mechanisms in improving the model’s classification capabilities. Conclusion: The integration of multiple attention mechanisms enhances the model’s ability to accurately classify LSS in MRI images, demonstrating its potential as a tool for automated diagnosis. This study paves the way for future research in applying attention mechanisms to the automated diagnosis of lumbar spinal stenosis and other complex spinal conditions.

## 1. Introduction

Lumbar spinal stenosis is one of the major disabling factors for low back pain in the elderly, and it is estimated to affect approximately 103 million people worldwide [1]. The prevalence of LSS and low back pain in low- and middle-income countries, especially in the elderly population, is approximately 3.5 times higher than that in high-income countries, where LSS has gradually become one of the leading causes of spine-related surgery, a figure that has attracted widespread attention from clinical researchers [2,3]. However, differences in prevalence data may reflect the unequal distribution of healthcare resources in different regions of the globe as well as differences in diagnostic criteria. Studies have shown that the prevalence of LSS in adults ranges between 11% and 39% and shows a positive correlation with increasing age [4]. Although LSS is indeed closely associated with spinal degeneration, including degeneration of the joints, intervertebral discs, and ligamentum flavum, its specific pathomechanisms need to be further explored [5]. Anatomical studies have shown that LSS can affect the central canal, lateral saphenous fossa, and intervertebral foramina. Clinical manifestations vary from person to person, with most patients experiencing pain in the lower lumbar region with discomfort in the buttocks and legs, and pain radiating to the calves and feet in some cases, especially when walking, while symptoms are relieved when sitting or bending over [6,7]. A cohort study noted that the L4-5 intervertebral space was the most involved site in LSS [8].

Although X-rays are often used as a screening tool due to their low cost, simplicity, and widespread clinical use, their value in the diagnosis of LSS is relatively limited [9]. In contrast, CT is able to demonstrate degenerative, erosive, and destructive changes in articular synovial joints more clearly and has advantages in the diagnosis of disc pathology [10]. However, MRI has become the imaging tool of choice for the evaluation of LSS due to its excellent soft-tissue contrast [11]. MRI not only provides high-resolution images of the spine and surrounding soft tissues, but also noninvasively demonstrates the detailed structures of the intervertebral discs, nerve roots, and spinal cord. Compared with other imaging techniques, MRI performs particularly well in the identification of early lesions and the assessment of complex anatomical structures, significantly improving the diagnostic accuracy for LSS [12]. In addition, MRI can also dynamically assess the blood flow and inflammation in the lesion area, providing an important basis for the development of individualized treatment plans. However, accurate interpretation of MRI results requires radiologists to have high levels of professional skill and experience, especially under a heavy workload, and the diagnostic accuracy may be somewhat challenged [13].

The dural sac cross-sectional area in T2-weighted axial MRI images of the lumbar spine is one of the commonly used imaging indices for the diagnosis of spinal stenosis; however, this method is highly subjective [14]. The grading system relies on the physician’s judgment, and the need to analyze the images of each disc segment one by one results in a time-consuming reading process for the imaging physician.

Early researchers tried to optimize the diagnostic process for lumbar spinal stenosis through traditional image feature extraction techniques and machine learning algorithms. However, in the face of complex anatomical structures and high individual variability, these methods still have obvious limitations that make it difficult to meet the practical needs of clinical applications.

For example, Koompairojn et al. [15] developed a computationally assisted system to segment MRI images using an active appearance modeling (AAM) technique by using T2-weighted axial views as inputs and ultimately using multilayer perceptrons for diagnosis. They obtained 92.66% accuracy on a dataset of 50 subjects. Koh et al. [16] proposed a diagnostic method for lumbar spinal stenosis based on magnetic resonance myelography images by segmenting the dural sac through binarization and edge detection techniques combined with a two-level classifier and achieved 91.3% diagnostic accuracy. Ruiz-Espana et al. [17], on the other hand, used a signal intensity segmentation and B-spline curve-fitting techniques to quantitatively assess the dural sac diameter ratio, yielding 70% sensitivity and 81.7% specificity for the classification and quantitative diagnosis of spinal stenosis.

With the rapid development of deep learning technology, convolutional neural networks (CNNs) have been widely adopted for medical imaging tasks, improving diagnostic accuracy and efficiency across various conditions. For instance, CNN-based methods have demonstrated success in MRI-based classifications, such as for brain tumors, Alzheimer’s disease, and IDH mutation status, demonstrating the versatility of these networks across different medical imaging tasks [18,19,20,21,22]. In the context of spinal disorders, particularly lumbar spinal stenosis (LSS), convolutional neural networks (CNNs) have also been widely used for detection and classification, with the expectation that they could improve the accuracy and efficiency of diagnosis. For example, Jamaludin et al. [23] developed a multi-task classification framework based on VGG-M for automatic prediction and localization of pathological features in spinal MRI, and the classification accuracy of intervertebral disc stenosis reached 87.8%. Han et al. [24] proposed a deep multi-scale multi-task learning network (DMML-Net) for lumbar neural foraminal stenosis automated diagnosis, which achieved an average accuracy of 0.845 on an MRI dataset of 200 patients, and performed well, especially in the diagnosis of neural foraminal stenosis. Lu et al. [25] designed a multi-input, multi-task, multi-class CNN model combining axial and sagittal MRI data, which achieved an accuracy of 80.4% in the classification of central spinal canal stenosis. A study by Won et al. [26] further validated the potential of deep learning models in spinal stenosis classification by training a CNN classifier that was highly consistent with expert diagnosis and demonstrated significant advantages in reducing diagnosis time and improving the reproducibility of results.

In recent years, the attention mechanism has gradually become a research hotspot in the field of medical image analysis due to its ability to dynamically adjust the model’s focus on input features [27,28]. Compared with traditional convolutional operations, the attention mechanism shows unique advantages in capturing complex anatomical structures and local features, especially in scenarios dealing with long-range dependencies and multi-scale information, demonstrating its potential ability to improve classification accuracy and model robustness [29]. However, despite significant progress in the application of attention mechanisms in other medical image tasks, such as tumor detection and organ segmentation, its use in the classification of lumbar degenerative diseases remains relatively limited. On the one hand, perhaps this is due to the longstanding lack of large-scale, high-quality lumbar spine MRI datasets, which limits further exploration in this area. On the other hand, the complexity of lumbar degenerative diseases themselves may also make the development of models based on attention mechanisms an additional challenge. However, in May 2024, the Radiological Society of North America (RSNA) and the American Society of Neuroradiology (ASNR) made publicly available a multicenter MRI dataset of lumbar degenerative disorders, which provides new opportunities for relevant research, although studies utilizing this dataset remain limited.

Despite the use of attention mechanisms in other areas, there remain several challenges in applying these techniques to lumbar spinal stenosis classification, leaving key research gaps:

- There is a lack of studies applying attention mechanisms specifically to lumbar degenerative disease classification.
- Existing models struggle with capturing complex anatomical details in MRI images of LSS.
- The application of multiple attention mechanisms, particularly combining different attention modules to improve accuracy, in lumbar spinal stenosis classification has not been fully explored.

To directly address these research gaps, this study proposes an innovative approach combining convolutional neural networks with multiple attention mechanisms. Specifically, we plan to introduce the Multi-Headed Self-Attention Module (MHSAM), Slot Attention Module (SAM), and Channel and Spatial Attention Module (CBAM) for MRI image classification of lumbar spinal stenosis. With this integrated approach, we aim to improve the accuracy of image classification, enhance the robustness of the model in processing complex medical images, and promote the development of automation and precision in the diagnostic process for lumbar spinal stenosis.

The main contributions of this study are reflected in the following aspects:

- A convolutional neural network architecture incorporating multiple attention mechanisms is proposed to significantly improve the classification accuracy for lumbar spine degenerative diseases.
- The key role of the attentional mechanisms in feature selection and global information capture is systematically verified through ablation experiments.
- The experimental results show that the proposed model outperforms the best existing model in several evaluation metrics.

The paper is structured as follows: Section 2 describes in detail the datasets, methods, and optimization techniques used. Section 3 presents the experimental results and analyzes them in comparison with other benchmark models. Section 4 discusses the experimental results in depth and evaluates the strengths and limitations of the model. Finally, Section 5 summarizes the main findings of the study and looks forward to future research directions.

## 2. Materials and Methods

**Figure 1:**
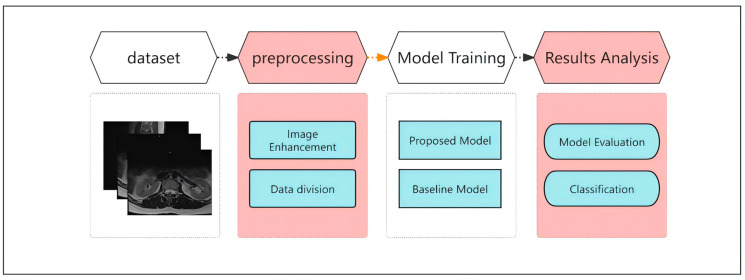
Workflow of the MRI image classification system for lumbar spinal stenosis.

Figure 1 presents the overall flow of this study, covering dataset acquisition, preprocessing, model training, and result analysis. First, we processed a large-scale standardized MRI dataset from multiple institutions. This dataset provides exhaustive imaging data for the study of lumbar degenerative spine lesions, and underwent rigorous preprocessing to ensure the reliability and accuracy of subsequent model training. The following sections describe the composition of the dataset and the processing methods in detail.

### 2.1. Description of the Dataset

This study utilized a subset of a multi-institutional MRI dataset, specifically designed to assist in the classification of lumbar degenerative diseases through magnetic resonance imaging (MRI). The dataset [30], jointly collected by the Radiological Society of North America (RSNA) and the American Society of Neuroradiology (ASNR), includes MRI scans from eight healthcare institutions across five continents. This diverse dataset provides a standardized foundation for classifying lumbar spine disorders and facilitates diagnostic concordance between healthcare institutions globally.

The dataset includes three primary MRI sequences, sagittal T2/STIR, sagittal T1, and axial T2, each capturing distinct pathological characteristics at different levels of the lumbar spine. These sequences comprehensively cover key lumbar spine pathologies, enabling more accurate diagnostic modeling.

Since the original MRI images were stored in DICOM format and had varying resolutions depending on the imaging equipment and settings from different institutions, preprocessing was necessary to ensure consistency across all images. For this study, we focused on the L4-L5 disc region, a critical area for diagnosing five types of degenerative conditions: spinal canal stenosis, left and right neural foraminal narrowing, and left and right subarticular stenosis.

Regions of interest (ROIs) were extracted from each MRI sequence to specifically target the L4-L5 disc area. After extraction, the images were resampled to 224 × 224 pixels using bilinear interpolation. For each of the five conditions—spinal canal stenosis, left and right neural foraminal narrowing, and left and right subarticular stenosis—a total of 1632 images were included, resulting in a dataset of 8160 images. Each lesion was categorized as either “normal/mild” or “severe” based on its severity, ensuring that the dataset adequately represents both common and severe cases for effective diagnostic modeling. The frequency distribution of different types of lumbar spinal stenosis is presented in Table 1.

### 2.2. Dataset Preprocessing Methods

**Figure 2:**
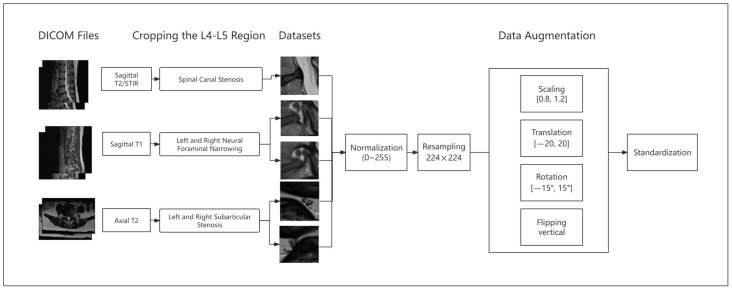
Workflow of dataset preprocessing.

To ensure that the model can efficiently process the MRI image data and improve its generalization ability, a multi-step image preprocessing pipeline was adopted in this study, as illustrated in Figure 2. These steps included normalization, image resizing, data augmentation, and standardization. The design of these preprocessing steps was based on the established literature and the specific characteristics of the lumbar spinal stenosis (LSS) dataset, with experimental verification of their validity in improving the model’s performance.

#### 2.2.1. Image Normalization

Since the original MRI images from various institutions were provided in DICOM format with varying pixel intensity ranges, the first preprocessing step involved normalizing all image pixel values to the 0–255 range. This normalization step is crucial for standardizing image intensity, ensuring that the images are comparable across different acquisition devices and settings [31,32,33]. Normalization provides a consistent input format for the subsequent steps, helping to reduce data variability while maintaining the original image distribution.

#### 2.2.2. Image Resizing

After normalization, all images were resized to 224 × 224 pixels to conform to the input size requirement of the model architecture. This size was selected to balance computational efficiency [34,35] with the need to preserve key anatomical features in the lumbar spine, particularly the L4-L5 disc region where degenerative changes are observed. The bilinear interpolation method [36,37,38] was employed during this step to minimize information loss, and initial experiments confirmed that this resolution is adequate for diagnosing conditions such as spinal canal stenosis, neural foraminal narrowing, and subarticular stenosis.

#### 2.2.3. Data Augmentation

Considering the directionality of MRI images of lumbar spinal stenosis in terms of spatial features, in order to prevent the model from overfitting and to enhance its generalization ability, we implemented a variety of data enhancement strategies [39,40,41] in the training set. These enhancement operations included:

- Scaling: We randomly adjusted the scaling of the images in the range of [0.8, 1.2] to simulate variations at different imaging distances and scales, thereby enhancing the robustness of the model to size variations.
- Translation: The image was randomly panned over a range of [−20, 20] pixels to simulate slight changes in patient position to enhance the model’s adaptability to different imaging positions.
- Rotation: The random rotation angle was set in the range of [−15°, 15°] to enhance the model’s performance in response to non-standard shooting angles, especially its ability to cope with different angles of imaging.
- Flipping: Given that the LSS lesions in some patients exhibit top–bottom symmetry, randomized vertical flip manipulation was applied to increase data diversity and prevent the model from relying too heavily on information from a specific orientation.

These data enhancement strategies are based on the anatomical properties of lumbar spine MRI images and aim to improve the robustness and anti-interference ability of the model and ensure its generalization performance under different imaging conditions and lesion distributions. In addition, through data augmentation, we effectively alleviate the problem of uneven distribution of different categories of data in the training set, especially enhancing the model’s ability to recognize a few categories (e.g., severe stenosis).

#### 2.2.4. Image Standardization

Following data augmentation, all images underwent standardization. Each image channel was demeaned to centralize the data [42,43], improving the consistency of the inputs. Standardization helps align the data distribution with the assumptions of the neural network, which accelerates convergence during training and reduces variability between batches. This step contributes to model stability during both training and testing phases, facilitating improved performance on unseen data.

### 2.3. Proposed Architecture

**Figure 3:**
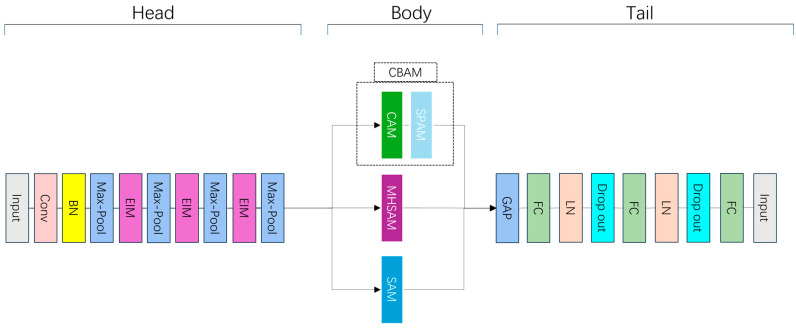
Overall architecture of the proposed model. The model consists of three major parts: the head, body, and tail modules. The head module includes convolutional layers (Conv), Batch Normalization (BN), and Enhanced Inception Modules (EIM) for feature extraction, followed by Max-Pooling (Max-Pool) layers to downsample the feature maps. The body module incorporates four attention mechanisms: the Channel Attention Module (CAM), Spatial Attention Module (SPAM), Multi-Head Self-Attention Module (MHSAM), and Slot Attention Module (SAM), which collectively enhance feature selection and improve classification performance. Additionally, the Convolutional Block Attention Module (CBAM) combines the Channel Attention Module and the Spatial Attention Module to refine features in both channel and spatial dimensions. The tail module applies Global Average Pooling (GAP), fully connected (FC) layers, Layer Normalization (LN), and Dropout to refine the final classification output.

**Figure 4:**
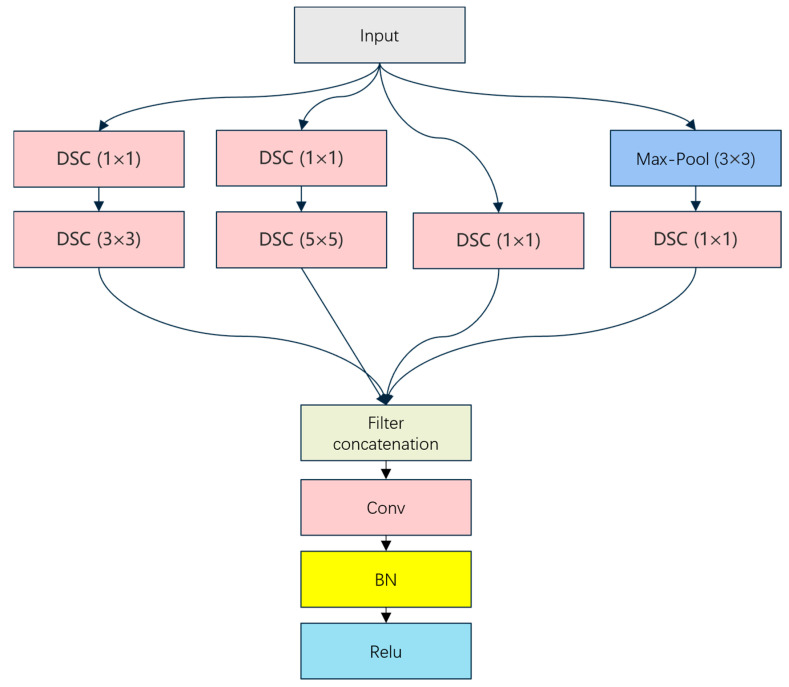
Structure of the Enhanced Inception Module. This module consists of multiple parallel paths for extracting features at various scales. Depth Separable Convolutions (DSC) of varying kernel sizes (1 × 1, 3 × 3, 5 × 5) are applied in parallel, along with a Max-Pooling (3 × 3) operation. The results from all branches are concatenated (Filter concatenation) before being passed through a 1 × 1 convolution, followed by Batch Normalization (BN) and a ReLU activation function. This structure allows for efficient multi-scale feature extraction while reducing computational complexity through depth-wise separable convolutions.

This study proposes an innovative model architecture incorporating deep learning techniques, combining convolutional neural networks (CNNs) and attentional mechanisms, for the automated classification of MRI images of lumbar degenerative diseases. Our model architecture is specifically divided into three major parts, the head module, the body module, and the tail module, which perform different functions. Figure 3 provides an overview of the overall architecture of the proposed model. Specifically, the head module is the starting part of the whole model, which is responsible for accepting data input and extracting low-level features. In detail, we first extract features from the image data through a 7 × 7 size convolutional kernel, then reduce the feature differences of the image data through Batch Normalization (BN), then we learn the feature model through the ReLU activation function, and the head module repeats this process many times to enable the model to extract the low-level features of the image data. In order to reduce computational complexity and retain key information, the head module introduces a Max-Pooling layer to downsample the spatial dimension of the feature map. In addition, to further enhance the ability to capture multi-scale features, the module integrates the Enhanced Inception Module (EIM), which enriches the details of feature extraction through convolutional kernels at different scales and Depth Separable Convolution (DSC) [44,45] while effectively reducing the computational overhead. The detailed structure of the Enhanced Inception Module is illustrated in Figure 4.

The body module is the core part of the model and contains three parallel sub-modules, namely the CBAM, the MHSAM, and the SAM. These modules optimize features from different dimensions to capture the complexity of global and local information. The outputs of the three branches are finally integrated through the Global Average Pooling (GAP) layer to provide high-level feature representations for the ensuing classification task.

The selection of these attention mechanisms was based on their complementary roles in MRI image classification, as demonstrated by our ablation studies. The MHSAM captures long-range dependencies and global context, which is essential for identifying subtle but significant features distributed across the image, particularly in conditions like spinal canal stenosis where global information is critical. The CBAM enhances the model’s ability to focus on important regions by refining features in both the channel and spatial dimensions, which plays a significant role in conditions such as neural foraminal narrowing, as reflected by the drop in accuracy and F1 score when the CBAM is removed. The SAM clusters related features, improving the extraction of complex anatomical structures relevant to lumbar spinal stenosis, which is particularly beneficial for subarticular stenosis, where its clustering ability aids in capturing the complex local structures.

Our preliminary experiments showed that the combination of these attention mechanisms provided the best balance between enhanced feature extraction and computational efficiency. Adding additional attention modules did not yield further significant improvements in accuracy. This chosen setup optimizes the model’s performance by focusing on complementary aspects of feature extraction, with each module enhancing the model’s ability to capture different aspects of lumbar spinal stenosis lesions while avoiding unnecessary complexity.

**Figure 5:**
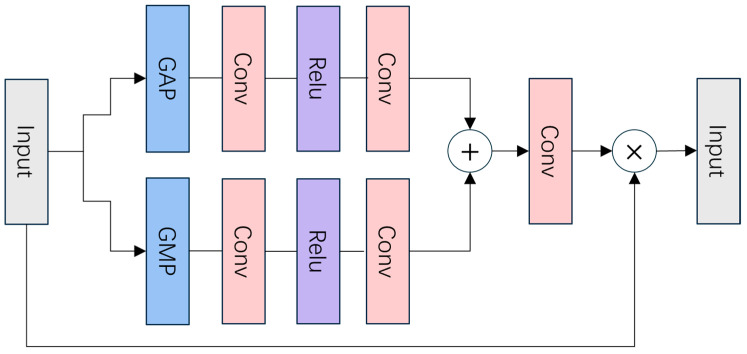
Structure of the Channel Attention Module (CAM). The CAM begins by applying Global Average Pooling (GAP) and Global Max Pooling (GMP) operations to the input feature map to capture channel-wise statistics. These pooled feature maps are then processed independently through convolutional layers followed by a ReLU activation function. The outputs from both pathways are summed and passed through another convolutional layer to generate the channel attention weights, which are multiplied with the original input feature map to refine it along the channel dimension, highlighting the most informative channels.

**Figure 6:**
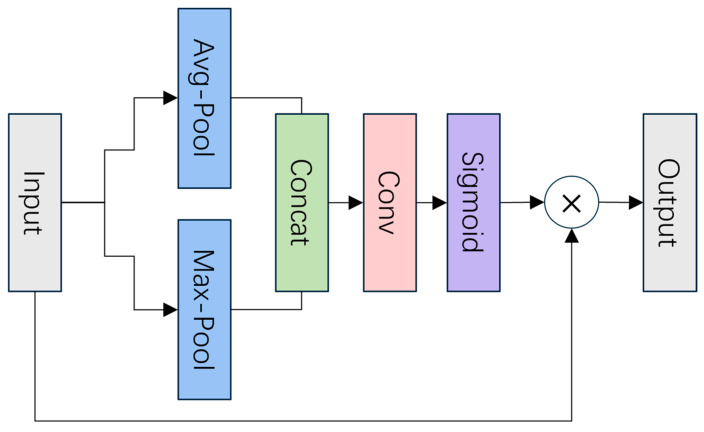
Structure of the Spatial Attention Module (SPAM). The SPAM module first computes the average and max-pooling across the channel dimension of the input feature map. The resulting two spatial feature maps are concatenated along the channel axis, forming a combined representation of spatial information. This concatenated feature map is then passed through a convolutional layer followed by a sigmoid activation to generate spatial attention weights. These weights are multiplied with the input feature map, focusing the model’s attention on the most relevant spatial regions, thus improving feature localization for subsequent layers.

The CBAM dynamically adjusts the model’s attention to different regions in the feature map by combining the channel and spatial attention mechanisms to enhance the recognition of lesion regions, as illustrated in Figure 5 and Figure 6. Specifically, the CBAM consists of the Channel Attention Module (CAM) shown in Figure 5, which focuses on channel-wise feature refinement, and the Spatial Attention Module (SPAM) shown in Figure 6, which refines the spatial dimensions of the feature map to further improve lesion localization. The detailed working process of CBAM is described in Algorithm 1. The channel attention weights are first generated through Global Average Pooling (GAP) and global max pooling (GMP), which are applied independently to each feature map within a batch. For each image in the batch, GAP and GMP are performed separately on the feature maps, ensuring that the pooling operations capture the important channel-wise features for that specific image without interference from other images in the batch. This is followed by the Spatial Attention Module, which further refines the feature selection by focusing on the spatial dimensions of the feature maps [46,47]. This process not only improves the sensitivity of the model to the lesion region in the image, but also effectively improves the relevance of feature extraction. The pseudo-code of the CBAM is as follows.
**Algorithm 1** Pseudo-code for the CBAM.**Require:**1:   *X*: Input feature map of dimension *C* × *H* × *W*2:   *r*: Reduction ratio for channel attention**Ensure:**   *X*′: Refined feature map after applying channel and spatial attention mechanism3: **Channel Attention Module:**4: Perform global average pooling:   *X*_avg_ = GAP(*X*)5: Perform global max pooling:   *X*_max_ = GMP(*X*)6: Apply two fully connected (FC) layers with ReLU activation between them:7:      *X*_fc1_ = ReLU(FC1(*X*_avg_))8:      *X*_fc2_ = ReLU(FC1(*X*_max_))9: Combine average and max-pooled outputs:10:      *M_c_* = *σ*(FC2(*X*_fc1_ + *X*_fc2_))11: Apply channel attention to input:12:      *X_c_* = *X* ⊗ *M_c_*13: **Spatial Attention Module:**14: Compute channel-wise average and max along the channel axis:15:      *X*_avg_ch_ = Mean(*X_c_*, dim = *C*)16:      *X*_max_ch_ = Max(*X_c_*, dim = *C*)17: Concatenate along the channel axis:18:       *X*cat = Concat(*X*avg_ch*, X*max_ch) 19: Apply a convolution layer:20:      *M_s_* = *σ*(Conv(*X*_cat_))21: Apply spatial attention to the input:22:      *X_s_* = *X_c_* ⊗ *M_s_*23: **return** *X*′ = *X_s_*

**Figure 7:**
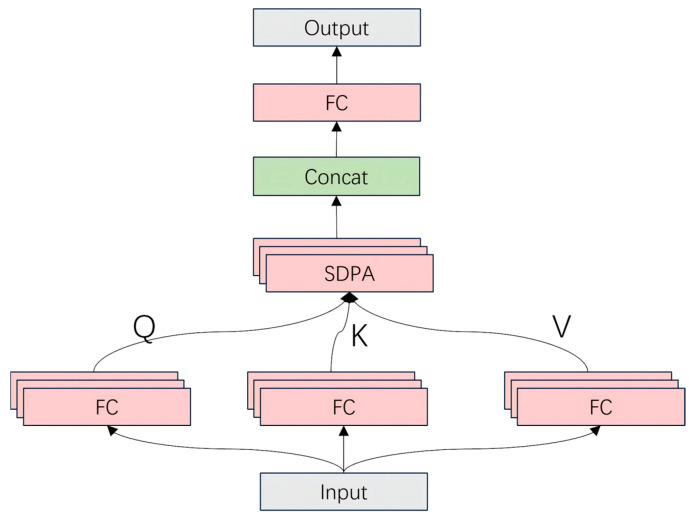
Structure of the MHSAM. The MHSAM employs multi-head self-attention to enhance the model’s ability to focus on different aspects of the input feature representation. The input feature map is first linearly projected into query (Q), key (K), and value (V) matrices. Each of these matrices is split into multiple heads, which allows the model to attend to information at different positions simultaneously. The scaled dot-product attention (SDPA) is computed for each head, capturing the relationships between different spatial locations in the feature map. Finally, the outputs from all heads are concatenated and transformed through a fully connected (FC) layer to generate the refined feature representation, which is passed on to subsequent layers for further processing.

The MHSAM strengthens the model’s ability to capture complex structural information by introducing the multi-head self-attention mechanism, as shown in Figure 7. The multi-head self-attention mechanism, detailed in Algorithm 2, can be executed in parallel on multiple attention heads, which effectively improves the model’s expressiveness in different feature dimensions [48,49], gives it a more global perspective, and is especially suitable for processing complex image data. The pseudo-code of the MHSAM is as follows.
**Algorithm 2** Pseudo-code for the MHSAM.**Require:**1:   *V, K, Q*: Input feature maps (values, keys, queries) of dimension *C* × *H* × *W*2:   *h*: Number of attention heads3:   *d*: Embedding dimension4: **Ensure:** Refined output feature map of dimension *C* × *H* × *W*5: **Reshape:**6:   Reshape *V, K, Q* to *N* × (*H* × *W*) × *C*7: **Apply linear transformations:**
8:   *V, K, Q* = Linear(*V*)*,* Linear(*K*)*,* Linear(*Q*)9: **Split into multiple heads:**10:  
$\mathrm{Reshape}\;V,\;K,\;Q\;\mathrm{into}\;N\times h\times \left(H\times W\right)\times \frac{d}{h}$
11: **Compute scaled dot-product attention:**12:   $\mathrm{Attention}=\mathrm{softmax}\left(\frac{Q\cdot K^{T}}{\sqrt{d/h}}\right)$
13: **Compute weighted sum of *V*:**
14:   *O* = Attention · *V*15: **Reshape output:**
16:   Reshape *O* back to *N* × (*H* × *W*) × *d*17: **Apply final linear transformation:**18:   *O* = Linear(*O*)19: **Reshape to original dimensions:**20:   Reshape *O* to *N* × *C* × *H* × *W*21: **return** *O*

The SAM, on the other hand, aggregates the input features by iteratively optimizing multiple slots (slots), which is particularly suitable for processing indeterminate length features and feature clustering tasks, as described in Algorithm 3. In scenarios with high uncertainty or complex and changing data features, the SAM demonstrates strong clustering ability and enables the model to flexibly cope with diverse inputs. The pseudo-code of the SAM is as follows.
**Algorithm 3** Pseudo-code for SAM **Require:**1:   *x*: Input feature map of dimension *B* × *C* × *H* × *W*2:   num slots: Number of slots3:   dim: Dimensionality of each slot**Ensure:** Final slot representation4: **Apply global average pooling to input feature map:**5:   *x*_avg_ = GlobalAvgPool(*x*) 6: **Initialize slots:**7:  
$S_{0}\;=\mu +\sigma \cdot \;N\left(0,\;1\right)$
8: **Iterative slot refinement:**9: **for**
*t* = 1 **to** iters **do**10:  Normalize slots: *S_t_* = Layer Normalization(*S_t_*_−1_)11:  
$\mathrm{Compute}\;\mathrm{attention}\;\mathrm{scores}:\;A_{t}=\mathrm{Softmax}\;\left(\frac{S_{t}\cdot x_{\mathrm{avg}}^{T}}{\sqrt{\dim}}\right)$
12:  Update slots: *S_t_* = *S_t_* + MLP(*A_t_* · *x*_avg_)13: **end for**14: **Reshape slots to match output dimensions.**
      **return** Final slot representation

Finally, the tail module is responsible for categorizing the high-level features extracted from the body module. This module consists of multiple fully connected layers, and maps the extracted high-dimensional features to the target classification space through layer-by-layer feature projection and normalization operations. In order to prevent overfitting, Dropout layers are introduced between modules to improve the generalization ability of the model. The final output is the probability distribution of each classification.

All in all, the model architecture effectively combines the advantages of convolutional neural networks in feature extraction and the powerful ability of multiple attention mechanisms in global information capture and feature selection. Through the organic combination of these modules, the model is able to accurately process complex features in MRI images, highlight key information, and achieve excellent classification performance. This architecture provides a powerful tool for automated medical imaging diagnosis and shows a wide range of application potential.

### 2.4. Optimization Techniques

During the training process of deep learning models, in order to ensure that the models have good convergence, robustness, and generalization ability, we employ a series of optimization techniques to deal with common challenges in training, such as overfitting and gradient vanishing problems. Optimization strategies such as Dropout, Layer Normalization (LayerNorm), Batch Normalization, and the Adam optimizer are introduced in this study. The application of these techniques not only improves the training effect of the model, but also lays a solid foundation for subsequent experiments. However, although these techniques perform well in most scenarios, there is still some uncertainty about their actual effectiveness in specific tasks. Next, we will introduce the principles of each technique in detail and discuss its specific performance in the experiments.

#### 2.4.1. Dropout

Dropout is a commonly used regularization method, and its core idea is to enhance the generalization ability of the model by introducing noise during the forward propagation process of model training [50]. In each iteration of the training, the Dropout layer randomly discards some of the neuron connections, which makes the weight update of the neural network no longer rely on the hidden relationships between local nodes, and thus effectively prevents the model from over-relying on certain features. However, although Dropout reduces the risk of overfitting, overuse can also lead to information loss, especially in the case of more complex models or smaller datasets. This process is somewhat equivalent to averaging the training parameters of multiple neural networks, thus reducing the occurrence of extreme cases. In this study, we introduced the Dropout operation after the fully connected layer and set the parameter to 0.5. By this method of randomly dropping some neurons, the generalization ability of the model was significantly improved.

#### 2.4.2. Layer Normalization

Batch Normalization is for the case of mini-batch training, and, in order to perform normalization even when there is only one training sample, the Layer Normalization technique is proposed. Unlike Batch Normalization, which relies on small batches of data for normalization, LayerNorm normalizes the features of each sample independently, which is especially critical for small batches of training or testing phases [33,51]. Specifically, by normalizing the mean and standard deviation of each feature, Layer Normalization not only accelerates the convergence speed of the network, but also mitigates the instability of the gradient to some extent. In addition, it can also enhance the robustness of the model in the face of different data distributions, thus improving the performance of the model in practical applications.

#### 2.4.3. Batch Normalization

As the depth of the network increases, the problem of vanishing or exploding gradients becomes more significant in convolutional neural networks. Batch Normalization (BN) can effectively alleviate this problem by normalizing each batch of data after the convolutional layer. Its main role is to accelerate the convergence process of the model by stabilizing the data distribution and reducing the internal covariate bias [52,53]. In this study, the BN layer is introduced to the head part of the convolution operation after ensuring that the features input to the nonlinear activation function before remain within a reasonable distribution, and this design significantly improves the training effect of the deep network.

#### 2.4.4. Adam Optimizer

The Adam optimizer, the main optimization algorithm in this study, has been widely used in various deep learning tasks thanks to its adaptive momentum estimation property. By combining the advantages of Momentum and RMSProp, the Adam optimizer is able to accelerate the convergence of the model through exponentially weighted averaging of the accumulated gradients while dynamically adjusting the learning rate for each parameter to solve the problem of gradient asynchrony [54,55]. Specifically, Adam adaptively adjusts the step size at each update by calculating the first-order momentum and second-order momentum of the gradient, thus demonstrating significant performance advantages in the case of sparse or noisy gradients. The optimizer not only reduces gradient oscillations, but also improves the overall stability during training. In this study, the initial learning rate is set to 0.001, and, to further enhance the generalization ability of the model and avoid overfitting, we also introduce Early Stopping, which terminates training when the performance of the validation set is no longer improving.

## 3. Experimental Results

### 3.1. Evaluation Metrics

The core goal of our experiments was to evaluate the performance of our model in the task of categorizing lumbar degenerative diseases, so we used a variety of evaluation metrics. Specifically, we used metrics such as accuracy, precision, recall, and F1 score. The following is a detailed description.

- Accuracy reflects the percentage of correct predictions made by the model over the entire dataset. It is calculated by the following formula:

$$\mathrm{Accuracy}\;=\frac{TP+TN}{TP+TN+FP+FN}$$

where TP stands for true cases, TN for true-negative cases, FP for false-positive cases, and FN for false-negative cases.

- Precision represents the proportion of all samples predicted by the model to be in the positive category that are actually in the positive category. The formula is as follows:

$$\mathrm{Precision}\;=\frac{TP}{TP+FP}$$

- Recall, also known as the sensitivity or true-positive rate, measures the proportion of samples that the model correctly identifies as being in the positive category out of all samples that are actually in the positive category. It is calculated as follows:

$$\mathrm{Recall}\;=\frac{TP}{TP+FN}$$

- F1 score (F1-Score) is the reconciled average of precision and recall and is often used to balance the trade-off between the two and is particularly adaptable to the problem of category imbalance. The formula is as follows:

$$F1-\mathrm{Score}\;=2\times \frac{\;\mathrm{Precision}\;\times \;\mathrm{Recall}\;}{\;\mathrm{Precision}\;+\;\mathrm{Recall}\;}$$

With these metrics, we are able to more comprehensively assess the performance of the model in different aspects, thus validating its effectiveness in the task of lumbar degenerative disease classification and exhaustively comparing the results with those of other baseline models.

### 3.2. Experimental Platform and Training Process

The experimental platform of this study adopted the PyTorch 2.3.0 framework and combined several mainstream Python libraries, such as NumPy, Pandas, Matplotlib, and Scikit-learn, to support model construction and data processing. In terms of hardware, the experiments were performed with an NVIDIA RTX 3090 GPU (24 GB graphics memory, NVIDIA Corporation, Santa Clara, CA, USA), Intel Xeon Platinum 8362 CPU (32 cores, 2.80 GHz, Intel Corporation, Santa Clara, CA, USA), and 45 GB RAM (Samsung Electronics, Suwon, South Korea) to ensure the efficient operation of the model during the training and optimization process.

During the experiments, we divided the dataset into training and testing sets at a ratio of 80:20. In order to ensure the comparability of the results, all the experiments were conducted under the same hardware environment and parameter settings. In addition, an Early Stopping strategy was introduced in the experiments to avoid model overfitting.

### 3.3. Model Performance Comparison

**Figure 8:**
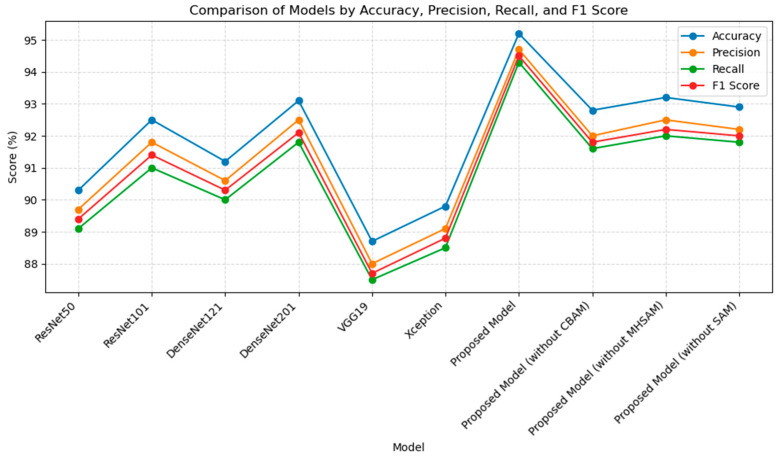
Comparison of the proposed model with other models.

As shown in Table 2 and Figure 8, the results show that the proposed model significantly outperforms existing models in all evaluation metrics, especially in the task of lumbar degenerative disease classification. Specifically, the model achieved 95.2% accuracy, 94.7% precision, 94.3% recall, and 94.5% F1 score. In comparison, the next best-performing model, the DenseNet201 model, achieved an accuracy of 93.1% and an F1 score of 92.1%. Our proposed model was improved by 2.1 percentage points in accuracy and 2.4 percentage points in F1 score. The main reason for this performance improvement may be the design of the multi-branch structure and the attention mechanism in the proposed model.

In the ResNet family, although ResNet101 performs well in terms of accuracy (92.5%) and F1 score (91.4%), its single residual linkage architecture is not as effective in capturing complex lesion features. DenseNet201 demonstrates an advantage in information flow with an accuracy of 93.1%, but it still lacks in capturing subtle features of complex images. This suggests that relying on high accuracy alone cannot completely solve all problems in medical image analysis tasks. In contrast, by introducing the multi-branching attention mechanism, our proposed model not only demonstrates superior ability in capturing details, but also significantly outperforms DenseNet201 and ResNet101 in terms of overall performance, particularly in handling more complex and varied medical images.

Additionally, the comparison of training times shows that the proposed model has a training time of 10,377 s, which is significantly shorter than DenseNet201’s 17,649 s, while still achieving superior results. This highlights the computational efficiency of the proposed model in addition to its accuracy improvements.

Table 3 further breaks down the performance across different lumbar spinal stenosis conditions. For example, for spinal canal stenosis, the proposed model achieves 95.2% accuracy, 94.7% precision, and 94.5% F1 score, whereas DenseNet201 achieves 93% accuracy and 92% F1 score. Similarly, for left neural foraminal narrowing, the proposed model achieves 95.4% accuracy, which is 2.2% higher than DenseNet201’s 93.2%. These results are consistent across all conditions, with the proposed model outperforming DenseNet201 across accuracy, precision, recall, and F1 score.

Table 4 expands on the performance across these conditions by examining the impact of individual attention modules. The analysis reveals that the CBAM shows a notable strength in identifying neural foraminal narrowing, improving the model’s sensitivity to local anatomical features. The MHSAM, on the other hand, excels in identifying spinal canal stenosis, capturing the global context through its multi-head attention mechanism. The SAM demonstrates its stability, particularly in identifying left subarticular stenosis and right subarticular stenosis, where its clustering ability optimizes complex feature extraction. Overall, the proposed model benefits from the synergy of all three modules, achieving balanced and superior performance across all stenosis conditions.

The results of the ablation experiments provide a more nuanced view. When we removed the CBAM, the performance of the model dropped significantly; the accuracy dropped to 92.8%, the precision decreased to 92.0%, and the F1 score correspondingly decreased to 91.8%. This not only highlights the critical role of the CBAM in feature capture but also emphasizes its non-negligible contribution to the overall model performance. Similarly, when the MHSAM was removed, the F1 score dropped significantly to 92.2%, although the accuracy dropped only slightly to 93.2%, which implies that the MHSAM is particularly important in improving the global information capture capability. When we removed the SAM, the accuracy of the model further decreased to 92.9%, and the F1 score also decreased to 92.0%, suggesting that the SAM plays an important role in local feature extraction.

Clearly, these results show that the model’s architectural innovations are key to its performance. Even though the removal of attention mechanisms lowers the performance, the proposed model still outperforms most existing models. The combination of the multi-branch structure and the attention mechanism greatly enhances the robustness and generalization ability of the model when dealing with complex medical image analysis tasks. Moreover, this design improves classification accuracy while significantly enhancing the model’s sensitivity to subtle lesion features.

**Figure 9:**
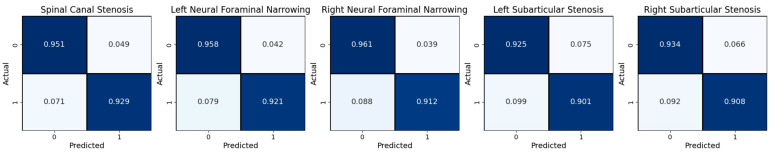
Confusion matrix for the DenseNet201 model. The matrix illustrates the classification performance of the DenseNet201 model, with 0 denoting normal or mild cases and 1 indicating severe cases. Furthermore, darker colors represent higher accuracy for the corresponding class.

**Figure 10:**
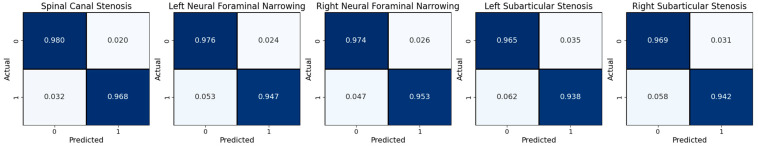
Confusion matrix for the proposed model. This matrix presents the classification outcomes for the proposed model, with 0 representing normal or mild cases and 1 denoting severe cases. Compared to the DenseNet201 model (Figure 9), the proposed model demonstrates improved accuracy, particularly in reducing false positives for severe cases, suggesting its potential for more reliable clinical application. Additionally, darker colors represent a higher level of accuracy in classification.

In addition, as shown by the confusion matrices in Figure 9 and Figure 10, the proposed model overall outperforms DenseNet201 in the classification of each category, especially in the identification of key lesions such as central canal stenosis and neural foraminal stenosis. It effectively reduces the cases in which mild lesions are misclassified as severe, indicating improved accuracy in differentiating lesion severity.

**Figure 11:**
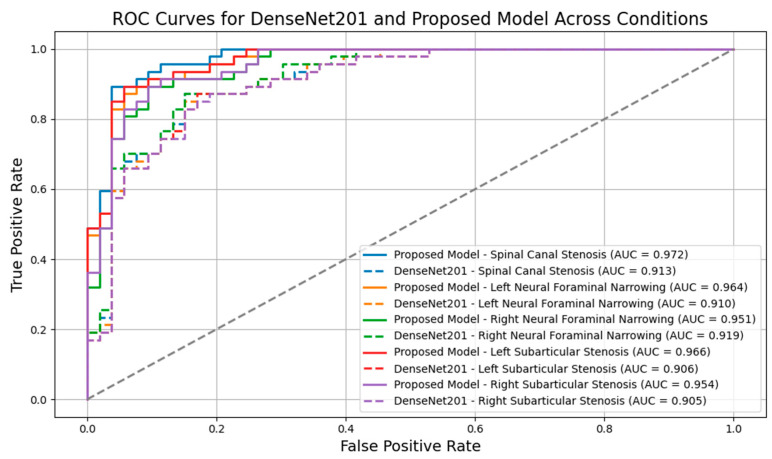
ROC curves for DenseNet201 and proposed model across conditions.

Moreover, the ROC curves in Figure 11 provide further insight into the model’s performance across different lumbar spinal stenosis conditions. The proposed model demonstrates superior Area Under the Curve (AUC) values in comparison to DenseNet201 across all conditions. For example, in the classification of spinal canal stenosis, the proposed model achieves an AUC of 0.972, while DenseNet201 achieves an AUC of 0.913. Similarly, for right neural foraminal narrowing, the proposed model’s AUC reaches 0.951, surpassing DenseNet201’s AUC of 0.919. These ROC curves indicate the proposed model’s better discrimination ability, further highlighting its reliability, particularly for clinical application, in distinguishing between mild and severe cases.

However, despite these improvements, the model’s performance on a few complex edge cases still needs refinement. In the future, larger-scale training data and the integration of multimodal information could potentially enhance its robustness and accuracy, ultimately providing a more reliable diagnostic tool for clinical settings.

### 3.4. Analysis of Misclassified Cases

**Figure 12:**
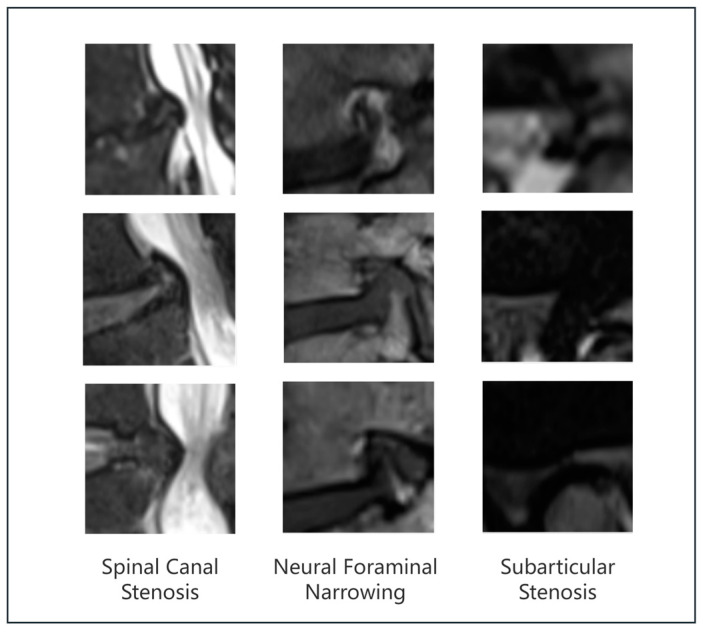
Misclassified MRI images in lumbar spinal stenosis diagnosis: severe cases incorrectly labeled as normal/mild.

Although the model proposed in this study has demonstrated excellent performance in most tasks, it encountered difficulties in some key cases, particularly when images from the “severe” category were misclassified as “normal/mild”. Figure 12 shows several examples of misclassified MRI images involving spinal canal stenosis, neural foraminal narrowing, and subarticular stenosis, highlighting the challenge of differentiating between severe and mild cases. We believe this is closely related to the inherent challenges in medical imaging diagnostic standards and data annotation. Firstly, the diagnostic standards for MRI images of different types of stenosis (such as spinal canal stenosis, neural foraminal narrowing, and subarticular stenosis) have not been fully unified. There are certain subjective differences in how institutions and doctors from different regions assess the severity of lesions [56,57,58]. The model’s higher error rate, especially in the classification of neural foraminal narrowing, may be related to the complex anatomy and blurred boundaries of this type of lesion, further increasing the classification difficulty. Additionally, there may also be anatomical differences among populations in different regions [59], which further complicates accurate classification.

Secondly, the dataset used in this study was collected from multiple medical institutions and regions, where variations in imaging equipment, imaging protocols, and diagnostic standards might exist, leading to inconsistencies in the annotation of lesion severity. Differences in the equipment also affect the model’s performance. Different MRI machines may vary in resolution, contrast, and signal-to-noise ratio, which can impact the observation and evaluation of subtle lesions. This is particularly the case when dealing with regions with rich anatomical details, such as those involved in neural foraminal narrowing and subarticular stenosis, where equipment differences may prevent the model from capturing sufficient detail.

Inconsistencies in annotation may further intensify in cases with unclear or hard-to-define lesion boundaries. Even among clinical professionals, there may sometimes be disagreement on the definition of the lesion region and the assessment of its severity [60,61], which contributes to the model’s declining performance in handling edge cases. This phenomenon is especially common in multi-institutional collaborative research, reflecting the challenges of ensuring consistent annotation in medical image analysis across institutions.

In future research, these challenges can be addressed by expanding the dataset size and unifying data annotation standards. Moreover, exploring multimodal data integration (e.g., combining CT images or clinical symptoms of patients) may enhance the model’s generalization ability and robustness when dealing with complex cases. Such an improvement based on multi-source information holds promise for increasing diagnostic accuracy and offers new directions for the clinical application of artificial intelligence in medical image analysis.

## 4. Discussion

This study introduces a novel convolutional neural network (CNN) model combined with multiple attention mechanisms to improve the classification accuracy of lumbar spinal stenosis (LSS) in MRI images. By integrating the Multi-Headed Self-Attention Module (MHSAM), Slot Attention Module (SAM), and Channel and Spatial Attention Module (CBAM), our approach enhances the model’s ability to capture the complex anatomical features in LSS, addressing the challenges seen in prior studies. In comparison to existing methods, such as that of Jamaludin et al. [62], who proposed a multi-task classification framework based on VGG-M with an accuracy of 87.8%, our method demonstrates superior performance with 95.2% accuracy. Jamaludin’s model, while efficient, struggled to capture complex global anatomical features due to its reliance on traditional feature extraction techniques. In contrast, our model’s use of attention mechanisms significantly enhances feature extraction, leading to improved classification performance, especially in differentiating subtle and complex lesion patterns. Table 5 provides a summary of these comparisons with other related studies.

Similarly, Han et al. [24] developed DMML-Net for diagnosing nerve root stenosis, achieving an accuracy of 0.845. However, their model encountered challenges with large-scale and diverse datasets, limiting its generalization capability in clinical applications. Our model addresses this limitation by leveraging a multi-branch architecture with multiple attention mechanisms, which not only enhances the robustness across diverse data but also improves the model’s adaptability to different MRI imaging conditions, as demonstrated by our experimental results.

Lu et al. [25] employed a multi-input, multi-task CNN model, improving classification accuracy to 80.4% for central canal stenosis and 78.1% for foraminal stenosis. While their approach demonstrated progress, the model was still limited in differentiating complex features, particularly for intervertebral foraminal stenosis. In comparison, our model’s incorporation of the CBAM and SAM allows for more effective feature selection and local–global information integration, resulting in higher classification accuracy across all stenosis types. For example, our model achieved 95.4% accuracy for left neural foraminal narrowing, a significant improvement over previous methods.

Won et al. [26] trained a CNN classifier on 542 axial MRI images and achieved accuracies between 77.9% and 83%, highly dependent on expert-labeled data. Our method, by contrast, not only surpasses these accuracy levels but also demonstrates more consistent performance on a larger, more diverse dataset. Furthermore, the multi-branch attention mechanism in our model allows it to be less reliant on manual annotations, providing greater potential for scaling to larger datasets with less expert involvement.

Hallinan et al. [63] conducted a more in-depth study on the consistency between deep learning models and radiologists. Their model, which simultaneously assessed central canal, lateral saphenous fossa, and neural foramen stenosis, achieved a Gwet κ value of 0.96 for central canal stenosis, demonstrating high consistency with expert annotations. However, their model performed less well in classifying neural foramen stenosis, with a Gwet κ value of 0.89, indicating limitations in capturing the complex anatomical features associated with this condition. In comparison, our model, incorporating multiple attention mechanisms, such as the CBAM, MHSAM, and SAM, enhances feature extraction in both the global and local dimensions, which improves classification performance, particularly in complex conditions like neural foramen stenosis. This integration leads to more accurate results, as reflected in the higher F1 score achieved by our model.

Natalia et al. [64] used a transfer learning technique to build a model based on Inception-ResNetv2, showing that classification using T2-weighted images significantly outperformed T1-weighted images. While this study demonstrated the benefits of migration learning, particularly in reducing training time and resource requirements, it still faced limitations in handling complex lesion classifications. These limitations may stem from the general transfer learning framework not being fully optimized for the nuances of lumbar spinal conditions. In contrast, our proposed model is specifically designed for lumbar spinal stenosis, leveraging attention mechanisms like the CBAM and SAM to capture the detailed structural features of LSS more effectively. This allows our model to achieve superior classification performance without relying heavily on migration learning techniques, ensuring better accuracy in complex medical imaging tasks.

Su et al. [65] developed a multi-task classification model based on ResNet-50, with classification accuracies ranging from 81.21% to 86.99% on lumbar disc herniation, central spinal stenosis, and nerve root compression. However, their model’s performance was constrained by the diversity and complexity of the dataset. Similarly, Altun et al. [66] proposed a VGG16-based model, achieving an accuracy of 87.70%, but the shallow nature of VGG16 limited its ability to capture the complex anatomical features of LSS. Our model, in comparison, surpasses these limitations by integrating attention mechanisms that allow for more precise feature selection, leading to improved performance across various LSS conditions.

Bharadwaj et al. [67] classified central canal stenosis, foraminal stenosis, and small joint lesions using V-Net and Big Transfer (BiT) models, achieving AUROCs of 0.94 and 0.92, respectively. Although these results were encouraging, the high computational complexity of their models makes them less feasible for resource-limited clinical settings. In contrast, our proposed model achieves similar or superior performance while maintaining computational efficiency, as evidenced by the significantly lower training time compared to that of DenseNet201 and other models. This balance between accuracy and efficiency is further demonstrated through our ablation experiments, which highlight the critical role of attention mechanisms in enhancing the model’s performance without adding unnecessary computational overhead.

Shahzadi et al. [68] reported accuracies of 97.01% and 97.71% on multi-ROI and single-ROI datasets, respectively, through data augmentation and segmentation techniques. However, their reliance on a relatively small dataset and extensive data augmentation raises concerns about potential overfitting, which may mask the model’s true performance in more complex real-world clinical settings. Our study, by contrast, utilizes a large, multi-institutional dataset and employs careful preprocessing techniques to ensure the generalizability of our results. The proposed model is specifically designed to handle diverse and complex LSS cases, making it more robust and applicable to a wider range of clinical scenarios.

Compared with the aforementioned studies, our multi-branch convolutional neural network model, which integrates an MHSAM, SAM, and CBAM, significantly improves the accuracy and robustness of LSS classification. The ablation experiments conducted in this study highlight the contributions of each attention mechanism in enhancing the model’s feature extraction and classification capabilities. This approach not only outperforms traditional models like VGG16 and ResNet-50 but also provides a new technical pathway for automated, accurate, and computationally efficient diagnosis of LSS in clinical settings.

Although this study demonstrates promising advancements in LSS diagnosis, there are still several limitations that require further exploration. First, the MRI dataset used only covers specific lesions in the L4-L5 segments, which may limit the model’s generalization capability for other degenerative spinal conditions. Future research should consider expanding the dataset to include more types of spinal conditions and additional anatomical regions. Additionally, while our model performed well on high-performance GPUs, its higher computational complexity may be a challenge in resource-constrained clinical environments. Future work could explore lightweight model designs to address these challenges while maintaining high accuracy.

Furthermore, while the attention mechanisms (the MHSAM, SAM, and CBAM) have contributed to improved classification performance, their interpretability in terms of clinical relevance remains to be fully explored. Future work, in collaboration with radiologists and orthopedic specialists, will focus on visualizing these mechanisms to provide deeper insights into how attention modules align with the imaging-based grading of lumbar spinal stenosis, potentially enhancing the clinical applicability of the model.

Finally, the model’s stability and interpretability in practical clinical applications remain areas for future interdisciplinary research. By addressing these challenges, our study lays a solid foundation for the potential clinical application of deep learning models with attention mechanisms in medical image analysis, particularly for the diagnosis of LSS.

## 5. Conclusions

In this study, we introduced a convolutional neural network model that integrates multiple attention mechanisms to enhance the accuracy of the classification of lumbar spinal stenosis (LSS) using MRI images. The model significantly outperforms existing baseline models in terms of accuracy, precision, recall, and F1 score, demonstrating the effectiveness of incorporating attention mechanisms such as the Multi-Headed Self-Attention Module (MHSAM), Slot Attention Module (SAM), and Channel and Spatial Attention Module (CBAM). These mechanisms contribute to more refined feature extraction by capturing both global and local anatomical details, which is particularly beneficial in distinguishing between mild and severe cases of LSS. Additionally, the ablation experiments underscore the importance of each attention module in improving the model’s performance, making it a robust tool for handling complex medical images. Despite its strengths, the study does face some limitations. The dataset used is focused on MRI images of the L4-L5 region, which may limit the model’s generalizability to other spinal regions or degenerative conditions. Moreover, while the model achieves high classification accuracy, its computational complexity could pose challenges for deployment in resource-constrained clinical environments. Future work should consider developing lighter models that retain accuracy while being more feasible for use in such settings. Additionally, there is room for improvement in the model’s handling of severe cases as it occasionally misclassifies them as mild, likely due to the variability in MRI image quality and diagnostic standards across institutions. Addressing these challenges, possibly through the inclusion of multimodal data or further refinements to the model architecture, will enhance the model’s applicability in clinical settings. Overall, this study highlights the potential of deep learning models with attention mechanisms to advance automated LSS diagnosis, and future research should focus on improving generalization capabilities, reducing computational demands, and expanding the application of this model to a wider range of clinical scenarios.

## Figures and Tables

**Table 1:** Frequency distribution of different types of lumbar spinal stenosis.

Condition	Normal/Mild	Severe	Total
Spinal stenosis	1429	203	1632
Left neural foraminal narrowing	1517	115	1632
Right neural foraminal narrowing	1525	107	1632
Left subarticular stenosis	1243	389	1632
Right subarticular stenosis	1244	388	1632

**Table 2:** Comparison of the proposed model with other models.

Model	Accuracy (%)	Precision (%)	Recall (%)	F1 Score (%)	Training Time (Seconds)
ResNet50	90.3	89.7	89.1	89.4	13,245
ResNet101	92.5	91.8	91.0	91.4	17,575
DenseNet121	91.2	90.6	90.0	90.3	7038
DenseNet201	93.1	92.5	91.8	92.1	17,649
VGG19	88.7	88.0	87.5	87.7	27,492
Xception	89.8	89.1	88.5	88.8	18,071
Proposed Model	95.2	94.7	94.3	94.5	10,377
Proposed Model (without CBAM)	92.8	92.0	91.6	91.8	8762
Proposed Model (without MHSAM)	93.2	92.5	92.0	92.2	7983
Proposed Model (without SAM)	92.9	92.2	91.8	92.0	8471

**Table 3:** Performance comparison of DenseNet201 and proposed model across different lumbar spinal stenosis conditions.

Condition	Model	Accuracy (%)	Precision (%)	Recall (%)	F1 Score (%)
Spinal Canal Stenosis	DenseNet201	93	92.4	91.7	92.0
Spinal Canal Stenosis	Proposed Model	95.2	94.7	94.3	94.5
Left Neural Foraminal Narrowing	DenseNet201	93.2	92.6	91.9	92.2
Left Neural Foraminal Narrowing	Proposed Model	95.4	94.9	94.5	94.7
Right Neural Foraminal Narrowing	DenseNet201	93.3	92.7	92.1	92.4
Right Neural Foraminal Narrowing	Proposed Model	95.5	95.0	94.7	94.9
Left Subarticular Stenosis	DenseNet201	92.9	92.2	91.5	91.8
Left Subarticular Stenosis	Proposed Model	95.1	94.6	94.1	94.3
Right Subarticular Stenosis	DenseNet201	93.0	92.4	91.7	92.0
Right Subarticular Stenosis	Proposed Model	95.2	94.7	94.3	94.5

**Table 4:** Performance of attention modules on lumbar spinal stenosis classification across different conditions.

Condition	Model	Accuracy (%)	Precision (%)	Recall (%)	F1 Score (%)
Spinal Canal Stenosis	Proposed Model	95.2	94.7	94.3	94.5
	with CBAM	93.2	92.5	91.9	92.1
	with MHSAM	94.5	94.0	93.5	93.7
	with SAM	92.9	92.2	91.8	92.0
Left Neural Foraminal Narrowing	Proposed Model	95.4	94.9	94.5	94.7
	with CBAM	94.0	93.5	92.9	93.2
	with MHSAM	93.5	92.9	92.3	92.6
	with SAM	93.1	92.6	91.8	92.2
Right Neural Foraminal Narrowing	Proposed Model	95.5	95.0	94.7	94.9
	with CBAM	94.7	94.1	93.6	93.8
	with MHSAM	93.6	92.9	92.2	92.5
	with SAM	93.2	92.6	91.9	92.2
Left Subarticular Stenosis	Proposed Model	95.1	94.6	94.1	94.3
	with CBAM	93.3	92.7	92.1	92.4
	with MHSAM	93.0	92.4	91.7	92.0
	with SAM	94.2	93.7	93.3	93.5
Right Subarticular Stenosis	Proposed Model	95.2	94.7	94.3	94.5
	with CBAM	93.4	92.8	92.2	92.5
	with MHSAM	93.2	92.5	91.9	92.1
	with SAM	94.1	93.5	93.0	93.2

**Table 5:** Summary of research on lumbar spinal stenosis classification.

Author	Dataset Type	Classification Target	Model Type	Main Results
Jamaludin et al. [23]	Sagittal T2-weighted MRI	Central canal stenosis	VGG-M multi-task classification framework	Accuracy: 87.8%
Han et al. [24]	Sagittal T1/T2-weighted MRI	Central canal stenosis and foraminal stenosis	DMML-Net	Average classification accuracy: 0.845; recall for nerve root stenosis: 0.8
Lu et al. [25]	Axial and sagittal T2-weighted MRI	Central canal stenosis and foraminal stenosis	Multi-task CNN	Central canal classification accuracy: 80.4%; foraminal stenosis: 78.1%
Won et al. [26]	Axial T2-weighted MRI	Central canal stenosis	VGG network	Model–expert consistency: 77.9–83%
Hallinan et al. [63]	Axial T2-weighted and sagittal T1-weighted MRI	Central canal stenosis, subarticular stenosis, and foraminal stenosis	CNN	Central canal classification Gwet κ value: 0.96; foraminal stenosis Gwet κ value: 0.89
Natalia et al. [64]	T1 and T2-weighted MRI	Central canal stenosis	Inception-ResNetv2	F1 score for T2-weighted images: 0.93; for T1-weighted images: 0.90
Su et al. [65]	Axial T2-weighted MRI	Central canal stenosis and foraminal stenosis	ResNet-50	Classification accuracy: 86.99% (central canal); 81.21% (foraminal stenosis)
Altun et al. [66]	2D MRI	Spinal stenosis	VGG16	Highest accuracy: 87.7%
Bharadwaj et al. [67]	Axial T2-weighted MRI	Central canal stenosis and foraminal stenosis	Big Transfer (BiT)	Central canal AUROC: 0.94; foraminal stenosis AUROC: 0.92
Shahzadi et al. [68]	Axial T1/T2 MRI	Central canal stenosis and nerve root stenosis	CNN	Multi-ROI accuracy: 97.01%; single ROI: 97.71%

## Data Availability

Data sources are cited within the article.
